# Myocardial edema during chemotherapy for gynecologic malignancies: A cardiac magnetic resonance T2 mapping study

**DOI:** 10.3389/fonc.2022.961841

**Published:** 2022-10-03

**Authors:** Meng-Xi Yang, Qing-Li Li, Dan-Qing Wang, Lu Ye, Ke-Min Li, Xiao-Juan Lin, Xue-Sheng Li, Chuan Fu, Xin-Mao Ma, Xi Liu, Ru-Tie Yin, Zhi-Gang Yang, Ying-Kun Guo

**Affiliations:** ^1^ Department of Radiology, Key Laboratory of Obstetric & Gynecologic and Pediatric Diseases and Birth Defects of Ministry of Education, West China Second University Hospital, Sichuan University, Chengdu, China; ^2^ Department of Radiology, West China Hospital, Sichuan University, Chengdu, China; ^3^ Department of Radiology, Sichuan Cancer Hospital & Institute, Sichuan Cancer Center, School of Medicine, University of Electronic Science and Technology of China, Chengdu, China; ^4^ Department of Gynecology, Key Laboratory of Birth Defects and Related Diseases of Women and Children of Ministry of Education, West China Second University Hospital, Sichuan University, Chengdu, China; ^5^ Department of Ultrasound, Key Laboratory of Birth Defects and Related Diseases of Women and Children of Ministry of Education, West China Second University Hospital, Sichuan University, Chengdu, China; ^6^ Key Laboratory of Carcinogenesis and Translational Research (Ministry of Education), Department of Radiology, Peking University Cancer Hospital & Institute, Beijing, China

**Keywords:** gynecologic malignancies, chemotherapy, cardiac magnetic resonance (CMR), myocardial edema, T2 mapping MRI

## Abstract

**Objective:**

Myocardial edema is an early manifestation of chemotherapy-related myocardial injury. In this study, we used cardiac magnetic resonance (CMR) T2 mapping to assess myocardial edema and its changes during chemotherapy for gynecologic malignancies.

**Methods:**

We enrolled 73 patients receiving chemotherapy for gynecologic malignancies, whose the latest cycle was within one month before the beginning of this study, and 41 healthy volunteers. All participants underwent CMR imaging. Of the 73 patients, 35 completed CMR follow-up after a median interval of 6 (3.3 to 9.6) months. The CMR sequences included cardiac cine, T2 mapping, and late gadolinium enhancement.

**Results:**

Myocardial T2 was elevated in patients who were treated with chemotherapy compared with healthy volunteers [41ms (40ms to 43ms) vs. 41ms (39ms to 41ms), P = 0.030]. During follow-up, myocardial T2 rose further [40ms (39ms to 42ms) vs. 42.70 ± 2.92ms, P < 0.001]. Multivariate analysis showed that the number of chemotherapy cycles was associated with myocardial T2 elevation (β = 0.204, P = 0.029). After adjustment for other confounders, myocardial T2 elevation was independently associated with a decrease in left ventricular mass (β = −0.186; P = 0.024).

**Conclusion:**

In patients with gynecologic malignancies, myocardial edema developed with chemotherapy cycles increase, and was associated with left ventricular mass decrease. T2 mapping allows the assessment of myocardial edema and monitoring of its change during chemotherapy.

## Introduction

As survival of cancer improves, cardiovascular toxicity has become a major cancer treatment-related complication (1–5). Among several pathological changes that occur in the myocardium during chemotherapy, edema is considered an early manifestation of myocardial injury and forerunner to cardiac dysfunction and myocardial fibrosis (6–9). Cardiac magnetic resonance (CMR) T2-mapping techniques can characterize myocardial edema *in vivo* and potentially provide additional insights beyond functional assessment (3, 10, 11). Animal studies have shown that anthracycline chemotherapy causes the prolongation of the myocardial T2 derived from T2 mapping, and histological analysis has further revealed good correlation between myocardial T2 and myocardial water content (7, 8). Clinical studies in humans have also reported increase in myocardial T2 as a sensitive biomarker for myocardial involvement during chemotherapy (9).

Gynecologic malignancies remain a major cause of mortality and morbidity in women (12), and the chemotherapy regimens for those patients consist of a variety of drugs which leading to myocardial injury (13–17). Moreover, to control tumor progression, patients in advanced stages typically require long-term chemotherapy regimens, rendering them at higher risk of cardiovascular diseases. However, systematic studies focusing on cardiovascular toxicity related to chemotherapy for gynecologic malignancies are lacking. We therefore enrolled patients with gynecologic malignancies and sought to investigate changes in myocardial edema during chemotherapy using T2 mapping techniques.

## Methods

### Study participants

The clinical protocol adopted in this study was approved by the institutional ethics review board of our hospital and of the Chinese Clinical Trial Registry (ChiCTR-DDD-17013450). All participants provided written informed consent forms before enrollment. Patients from the Department of Tumor Radiation and Chemotherapy who were diagnosed with gynecologic malignancies were recruited. Inclusion criteria were (1) current or recent (within the preceding month) receipt of chemotherapy and (2) age between 18 and 75 years (18, 19). Exclusion criteria were (1) concomitant diagnosis of cardiovascular diseases (coronary artery disease, primary cardiomyopathy, valvular heart disease, congenital heart disease, and pericardial disease), (2) prior receipt of treatment for other diseases carrying the potential for myocardial toxicity, and (3) contraindications to CMR. Age-matched healthy female volunteers were simultaneously recruited as the control group that underwent the same imaging procedures.

### Laboratory examination

We obtained venous blood samples from patients 1 hour before CMR imaging to examine serologic markers of myocardial injury, including troponin I and creatine kinase.

### Image acquisition

The CMR examinations were performed using a 3.0T magnetic resonance imaging machine scanner (Magnetom Skyra, Siemens Healthcare, Inc., Erlangen, Germany). The CMR protocol consisted of cardiac cine, T2 mapping, and late gadolinium enhancement (LGE) imaging. Cine imaging used a balanced steady-state free-precession pulse sequence: echo time (TE) = 1.22 ms, temporal resolution (TR) = 39.34 ms, flip angle = 40°, slice thickness = 8 mm, matrix = 208 × 208 pixels, and field of view (FOV) = 340 × 284 mm^2^. T2 mapping images were acquired using the steady-state free-precession technique, and three single-shot images were acquired at different T2 preparation times (0 ms, 25 ms, and 55 ms). The detailed parameters were as follows: TE = 1.23 ms, TR = 38.34 ms, flip angle = 60°, slice thickness = 8 mm, matrix = 208 × 208 pixels, and FOV = 250 × 300 mm^2^ (20, 21). For LGE imaging, intravenous gadolinium contrast (0.2 mL/kg) was first administered, a segmented phase-sensitive inversion recovery sequence with turbo FLASH readout at 17–19 minutes post contrast was then performed (22, 23): TR = 1.44 ms, TE = 300 ms, flip angle = 40°, slice thickness = 4 mm, matrix = 84 × 176 pixels, FOV = 153 × 106 mm. T2 mapping images were obtained in the basal, middle, apical short-axis, and four-chamber planes; cine and LGE images were obtained in the two-chamber, three-chamber and four-chamber planes, and a continuous stack of short-axis planes with full left ventricular (LV) coverage.

### Image analysis

Two experienced radiologists separately conducted the image analysis using imaging postprocessing software (CVI42, Circle Cardiovascular Imaging, Calgary, Canada). The analysis of LV functional parameters was presented in previous studies (9). Myocardial T2 was measured on the basal, middle, and apical color T2 maps and then averaged as the global LV myocardial T2 (24). Local myocardial fibrosis was defined based on the acquired LGE images: After elimination of artifacts, any obvious patch of the myocardium observed on any image was accepted as local myocardial fibrosis (25). If disagreements arose, a consensus was reached by discussion between the two radiologists.

### Reproducibility

To assess intraobserver variability, one radiologist randomly measured myocardial T2 in 39 subjects twice within 1 week. The other radiologist, who was blinded to the first radiologist’s results, then reperformed the measurements.

### Statistical analysis

Statistical analysis was performed using IBM SPSS Statistics (version 20.0; IBM Corp., Armonk, NY, USA) and GraphPad Prism (version 7.00, San Diego, CA) software applications. Categorical or enumeration parameters were presented as numbers (percentages), whereas continuous variables were expressed as the mean ± standard deviation or as medians (interquartile range), as appropriate. The chi-square test was used to compare categorical or enumeration parameters. The independent *t*-test or the rank-sum test was used to compare continuous variables between patients and healthy volunteers, as appropriate. Parameters from the two CMR examinations, performed in 35 patients who completed CMR follow-up, were compared using the paired *t*-test or rank-sum test, as appropriate. Bivariate correlation analysis was performed using the Pearson’s or Spearman’s method, as appropriate. To identify the factors that were independently associated with myocardial T2 and LV mass, a multivariate linear regression model was constructed. Intraobserver and interobserver variabilities of myocardial T2 measurements were assessed using intraclass correlation coefficient. All tests were two tailed; *P* values < 0.05 were accepted as statistically significant.

## Results

### Baseline characteristics

**Figure 1:**
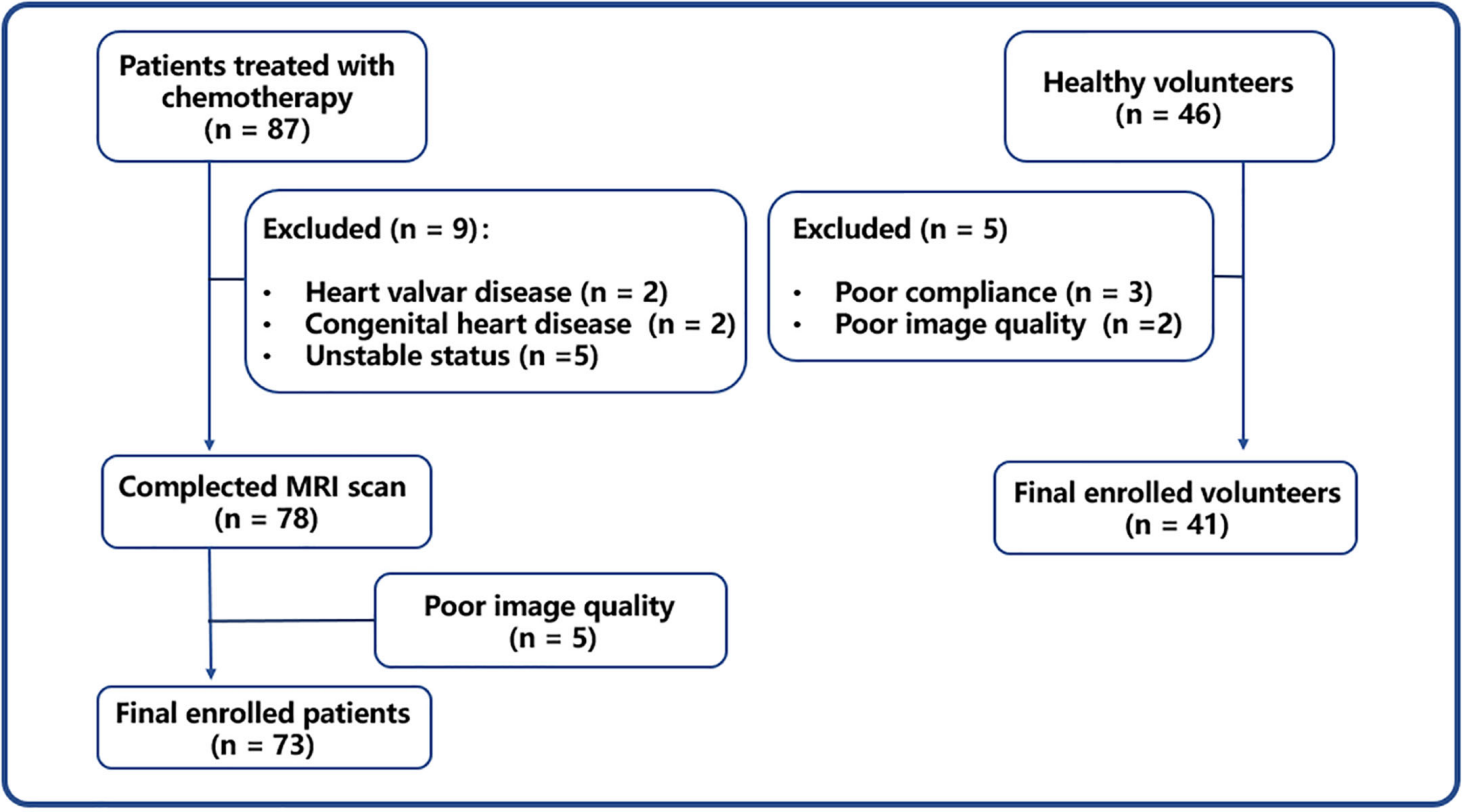
Study flowchart. CMR, cardiac magnetic resonance.

From September 2018 to December 2020, this study enrolled 73 patients treated with chemotherapy for gynecologic malignancies and 41 healthy volunteers (**Figure 1**). **Table 1** shows the baseline characteristics of patients and volunteers, and no statistically significant differences in baseline variables were found between the two groups (all *P* > 0.05). Moreover, no cardiovascular diseases or cardiovascular risk factors were detected in any of the enrolled volunteers.

**Table 1:** Baseline characteristics of patients and normal controls.

	Normal controls (n = 41)	Patients (n = 73)	P value
Age, year	45 (35 to 59.5)	50 (45 to 56)	0.192
Female, %	41 (100%)	73 (100%)	0.999
Hypertension, %	0 (0%)	6 (8.22%)	0.059
Diabetes, %	0 (0%)	3 (4.11%)	0.188
Hyperlipidemia, %	0 (0%)	0 (0%)	0.999
Smoker, %	0 (0%)	0 (0%)	0.999
Height, cm	157.4 ± 6.02	157.5 ± 5.46	0.938
Weight, kg	59.0 (51.5 to 60.5)	58.18 ± 9.61	0.394
Body-mass index, mm2	1.53 ± 0.15	1.55 ± 0.14	0.509
Receipt of cardiac medication			
Beta-blockers	_	4 (5.48%)	_
ARB	_	2 (2.74%)	_
ACEI	_	1 (1.37%)	_
Calcium antagonist	_	5 (6.85%)	_
Diuretics	_	1 (1.37%)	_
Heparin sodium	_	5 (6.85%)	_
Cancer type			
Ovarian cancer	_	45 (61.64%)	_
Carcinoma tubae	_	9 (12.33%)	_
Cervical cancer	_	16 (21.92%)	_
Endometrial cancer	_	3 (4.11%)	_
Cancer status and treatment			
Cancer stage	_	3 (2 to 3)	_
Cancer recurrence	_	24 (32.88%)	_
Operation (n, %)	_	72 (98.63%)	_
Pelvic radiation (n, %)	_	5 (6.85%)	_
Interval between first chemo and CMR scan, mon	_	5 (2 to 12)	_
Chemotherapy number	_	6 (3 to 12)	_
Chemotherapy regimen	_	1 (1 to 2)	_
Drug type number	_	2 (2 to 3)	_
Chemotherapy drug type (n, %)			
Paclitaxel + Platinum	_	64 (87.67%)	_
Anthracycline	_	12 (16.44%)	_
Cyclophosphamide/ifosfamide	_	17 (23.29%)	_
Bevacizumab	_	12 (16.44%)	_
Others	_	6 (8.22%)	_

BMI, body index; ARB, angiotensin receptor blockers; ACEI, angiotensin converting enzyme inhibitor. *P < 0.05 vs. normal control.

Among the 73 patients treated with chemotherapy, 6 (6/73, 8.22%) were complicated with hypertension and 3 (3/73, 4.11%) had diabetes. In addition, 6 patients (6/73, 8.22%) had a history of taking cardiac medication (**Table 1**). With regard to chemotherapy regimens, 64 patients (64/73, 87.67%) received a regimen consisting of paclitaxel plus platinum. The median interval between the first cycle of chemotherapy and CMR scan was 5 (2 to 12) months.

Of the 73 patients, 35 patients (35/73, 47.95%) completed the CMR follow-up. The interval between the two scans was 6 (3.3 to 9.6) months, and these patients completed 3 (2 to 5) cycles of chemotherapy during the interval.

### Alteration in LV morphology during chemotherapy

For LV functional parameters (**Table S1**), no significant difference was observed between the two groups in LV ejection fraction (LVEF) or LV end-systolic volume (all *P* > 0.05). However, the LV mass (43.80 ± 8.61 g/m^2^ vs. 46.90 ± 7.74 g/m^2^, *P* = 0.047) and LV end-diastolic volume (64.44 ± 12.94 ml/m^2^ vs. 69.58 ± 7.97 ml/m^2^, *P* = 0.038) were slightly lower in patients compared to healthy volunteers. Among 73 patients, 17 (17/73, 23.29%) were recorded as positive for LGE, as opposed to healthy volunteers who were all negative. At CMR follow-up in 35 patients (**Table S2**), a decrease in the LV mass was observed (47.17 ± 7.73 g/m^2^ vs. 44.33 ± 8.36 g/m^2^, *P* < 0.001), and three patients were newly recorded as positive for LGE (8/35, 22.86% initially vs. 11/35, 31.43% at follow-up; *P* = 0.250).

### Changes in myocardial T2 during chemotherapy

**Figure 2:**
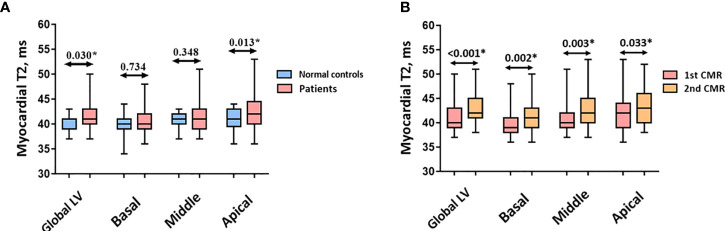
**(A)** Myocardial T2 between normal controls and patients. **(B)** Myocardial T2 between two CMR scans in patients. Abbreviations as in **Figure 1**. *p < 0.05 vs. normal control.

The global LV myocardial T2 was higher in patients than in healthy volunteers [41ms (40ms to 43ms) vs. 41ms (39ms to 41ms), P = 0.030; **Figure 2A**]. With regards to alterations in different slices, myocardial T2 in the apical slice was higher in patients than in healthy volunteers [42ms (40ms to 44.5ms) vs. 41ms (39.5ms to 43ms), *P* = 0.013], whereas myocardial T2 showed no difference in the middle and basal slices between the two groups (both *P* > 0.05).

**Figure 3:**
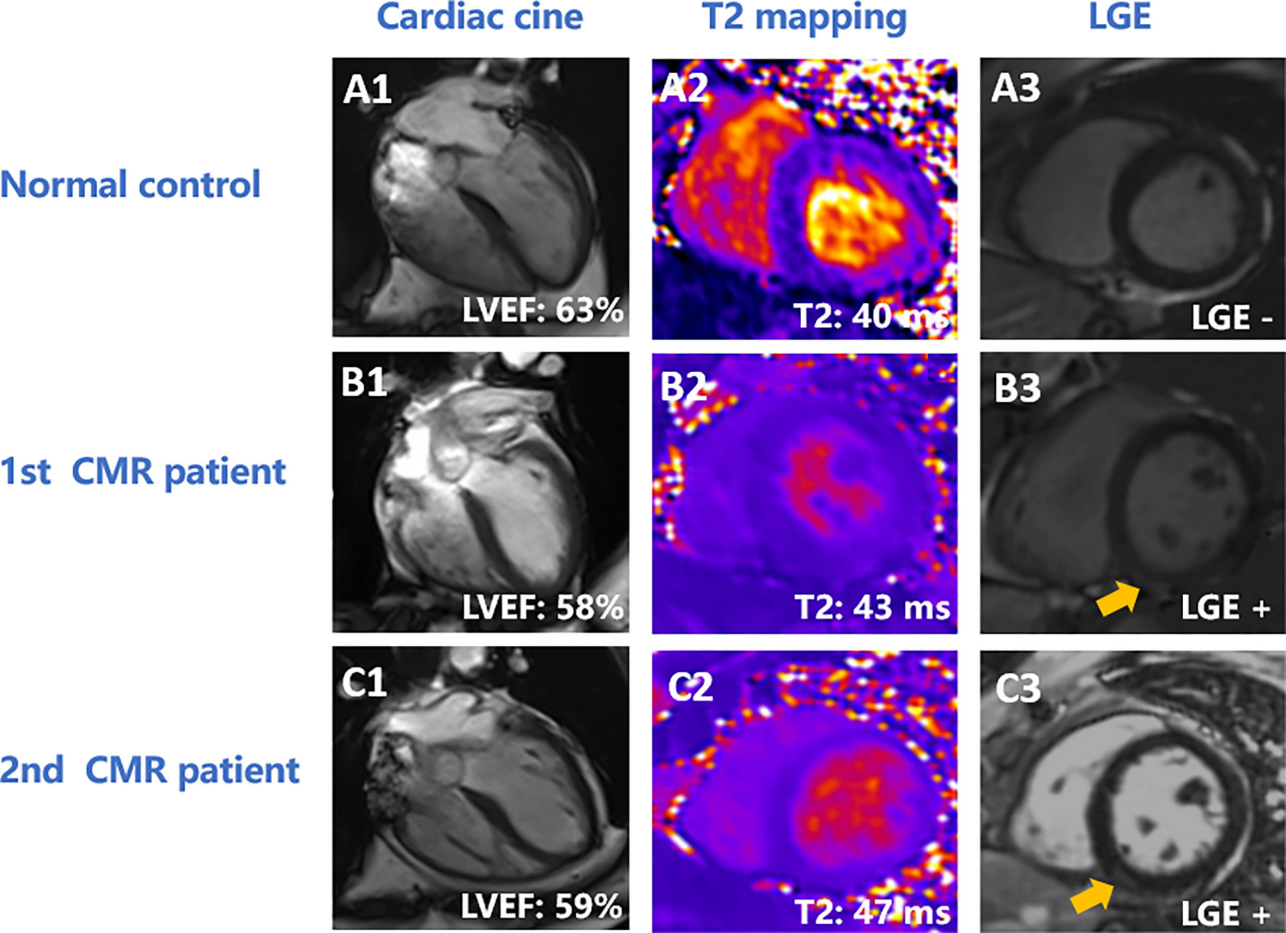
Representative CMR images of normal controls and patients. **(A)** A 58-year-old female healthy volunteer. LVEF was 63% **(A1)**, global LV myocardial T2 was 40 ms **(A2)**, and there was no enhancement on LGE **(A3)**. **(B, C)** A 44-year-old patient with ovarian cancer. The patient had undergone 12 cycles of paclitaxel plus platinum chemotherapy at the first CMR scan. LVEF was 58% **(B1)**, global LV T2 value was 43 ms **(B2)**, and had inferior enhancement (yellow arrow) on LGE **(B3)**. At 11-month follow-up, the patient had completed 15 cycles of paclitaxel plus platinum chemotherapy. LVEF was 59% **(C1)**, global LV myocardial T2 value was 47 ms **(C2)**, and had inferior enhancement (yellow arrow) **(C3)**. LVEF, left ventricular eject fraction; LGE, late gadolinium enhancement.

As the number of chemotherapy cycles increased, an elevation in global LV myocardial T2 was noted at follow-up [40ms (39ms to 42ms) vs. 42.70 ± 2.92ms, P < 0.001, **Figure 2B**]. Moreover, an increase in myocardial T2 in the apical, middle, and basal slices was observed (all P < 0.05). **Figure 3** presents the representative CMR images from healthy volunteers and patients.

### Association of myocardial T2 with myocardial injury biomarkers

In the 35 patients who completed CMR follow-up, the myocardial injury biomarkers tended to increase from baseline to follow-up imaging: creatine kinase [39u/L (15u/L to 41u/L) vs. 78.00u/L (58.25u/L to 95.00u/L), *P* < 0.001] and troponin I [0.006ug/L (0.006ug/L to 0.006ug/L) vs. 0.006 (0.006ug/L to 0.008ug/L), *P* < 0.001].However, no correlation was observed between myocardial T2 and creatine kinase (*r* = 0.152, *P* = 0.127) or troponin I (*r* = −0.046, *P* = 0.647).

### Influence of chemotherapy on myocardial T2 variation

Univariate analysis indicated that both the receipt of chemotherapy (r = 0.280, P = 0.001) and chemotherapy cycles received (r = 0.311, P < 0.001) positively correlated with myocardial T2 in all subjects. In model 1, after adjustment for chemotherapy drug types and time interval from first chemotherapy to CMR imaging, the number of chemotherapy cycles was associated with myocardial T2 (β = 0.462, P = 0.003). When Model 2 added age and clinical risk factors to the analysis, the number of chemotherapy cycles was also associated with an increase in myocardial T2 (β = 0.204, P = 0.029; **Table 2**).

**Table 2:** The association between chemotherapy and myocardial T2 variation.

Univariate analysis	R	P value
Chemotherapy	0.280	0.001*
Numbers of chemotherapy	0.311	<0.001*
**Multivariate analysis**	**β**	**P value**
Model 1: adjusting for time interval between first chemotherapy and CMR, and chemotherapy drugs (time interval, anthracycline, bevacizumab, and cyclophosphamide/ifosfamide)		
Chemotherapy	0.013	0.939
Numbers of chemotherapy	0.462	0.003*
Model 2: adjusting for clinical risk factors (age, hypertension, and diabetes)		
Chemotherapy	0.186	0.048*
Numbers of chemotherapy	0.204	0.029*

CMR, cardiac magnetic resonance. *P < 0.05.

### Association between myocardial T2 and LV mass

A negative association between myocardial T2 and LV mass was observed (*r* = −0.165, *P* = 0.045). In the multivariate analysis, after adjustment for CMR parameters and clinical risk factors (including age, hypertension and diabetes mellitus), myocardial T2 remained independently associated with a reduction in LV mass (β = −0.186, *P* = 0.024; **Table 3**).

**Table 3:** The risk factors of indexed LV mass by univariable and multivariable analysis.

	Univariable Analysis		Multivariable Analysis	
	R	P value	β	P value
Age	-0.159	0.053	-0.206	0.014*
Hypertension	-0.065	0.430	-0.033	0.697
Diabetes mellitus	0.156	0.058	0.253	0.002*
LVEF	-0.045	0.586	-0.027	0.734
Myocardial T2	-0.165	0.045*	-0.186	0.024*
LGE present	-0.083	0.317	0.005	0.953

LVEF, left ventricular eject fraction; LGE, late gadolinium enhancement. *P < 0.05.

### Reproducibility

With respect to the reproducibility of myocardial T2, the intraobserver and interobserver variability were measured at 0.955 (95% confidence interval [CI]: 0.914–0.976) and 0.904 (95% CI: 0.818–0.950), respectively.

## Discussion

This study enrolled patients treated with chemotherapy for gynecologic malignancies and used CMR T2 mapping to assess myocardial edema. The main findings were the following (1): Myocardial T2 was higher in patients than in healthy volunteers and increased as the chemotherapy cycles received increase during follow-up. (2) After adjustment for chemotherapy drug types and clinical characteristics, the number of chemotherapy cycles was associated with increased myocardial T2. (3) In the multivariate analysis including CMR and clinical risk factors, increased myocardial T2 was independently associated with lower LV mass. To the best of our knowledge, the present systematic study is the first to focus on myocardial edema associated with chemotherapy for gynecologic malignancies. Most patients included in this study underwent chemotherapy with paclitaxel plus platinum, a combination that is infrequently reported in literature. Our results showed that myocardial edema could be aggravated as chemotherapy continues and that myocardial edema is associated with a reduction in LV mass. Thus, the cardiac side effects of paclitaxel and platinum chemotherapy require clinical attention. Using CMR T2 mapping, clinicians can assess myocardial injury and track changes of myocardial edema during chemotherapy.

Chemotherapy-related cardiotoxicity develops because of a variety of physiological changes, and myocardial edema is an early manifestation (6–9). Quantitative assessment of myocardial edema through T2 mapping allows for the early detection of myocardial injury. In a rat model of anthracycline-induced myocardial edema, the prolongation of myocardial T2 was associated with an increase in myocardial water content, which appeared earlier than hemodynamic deterioration, LVEF decrease, and fibrous collagen deposition (7). In addition, Galán-Arriola observed the same change in large-animal models (8). In patients received with anthracyclines, Lustberg et al. demonstrated that myocardial T2 increased after the first cycle of chemotherapy, but LVEF and circumferential strain declined several months later (9). Similar to previous studies, our study found that myocardial T2 was higher in patients treated with chemotherapy for gynecological malignancies than in healthy volunteers, although no difference in LVEF was observed between the two groups. Moreover, in this study, the troponin I values of patients were within normal range and showed no correlation with myocardial T2, suggesting that the myocardial injury was in early stage, and troponin I was not significantly elevated. These results supported the advantage of T2 mapping for the early detection of myocardial injury during chemotherapy.

Additionally, our study revealed that the increase in myocardial T2 of patients was more pronounced in the apical slice than in the middle and basal slices. Although similar data haven’t been reported about the regional distribution of myocardial T2, published literatures have demonstrated the greatest motion impairments at heart apex in patients treated with chemotherapy (26, 27). Taken together, we speculated that apical myocardium is more vulnerable to chemotherapy. The potential causes might be the increased exposure of terminal circulation regions to chemotherapy drugs or the differential local activation of signal transduction (26), but the exact mechanism needs further exploration.

As reported in the previous studies, myocardial T2 varies with the individual, and is potentially influenced by factors such as age and sex (28–30). Longitudinal assessment of myocardial T2 during chemotherapy is helpful for recognizing change in myocardial edema, obviating the effects of other factors. In our patients, who completed the CMR follow-up, myocardial T2 tended to increase with the continuation of chemotherapy. Multivariate analysis demonstrated that the number of chemotherapy cycles was independently associated with an increase in myocardial T2. These results suggest that myocardial edema could be aggravated with an increase in chemotherapy cycles. Thus, patients treated with long-term chemotherapy are at an increased cardiac risk and require more attention. A study by Lustberg et al. supports our hypothesis, because those authors also observed a continuous increase in myocardial T2 during chemotherapy (9). On the other hand, myocardial edema subsides as chemotherapy ends or myocardial fibrosis develops. One study found a reduction in myocardial T2 at 12 months after the last chemotherapy cycle (25), and no difference was observed in myocardial T2 between cancer survivors after long-term chemotherapy and healthy individuals (31). The physiologic process of myocardial edema in patients undergoing chemotherapy for gynecologic malignancies requires further investigation with a longer follow-up period.

To date, clinical studies have focused mostly on the cardiotoxicity caused by anthracyclines and/or trastuzumab (7–9, 32–34); few studies have focused on cardiotoxicity caused by other chemotherapy drugs. Our study provides relevant data for other chemotherapy drugs used in gynecologic malignancies. Our patients received with various chemotherapy drugs, with the largest proportion receiving paclitaxel plus platinum regimens. Previous studies has reported that the cardiac implications of paclitaxel mostly manifest as myocardial ischemia or arrhythmia (35, 36), whereas platinum can lead to cardiac motion dysfunction (37). Our results suggest that paclitaxel plus platinum could potentially cause myocardial edema. Although the myocardial edema observed during chemotherapy was mild, myocardial injury could be aggravated as chemotherapy cycles increase. Paclitaxel and platinum are first-line drugs in a variety of tumors (38), more attention and further exploration of their cardiotoxicity is needed.

During chemotherapy for cancer, the left ventricle can undergo a series of morphological changes, and those changes have prognostic significance (3–5). Several studies reported that the receipt of chemotherapy was associated with a reduction in LV mass (39–42), which predicts for adverse cardiovascular events (41, 42). After adjustment for CMR and clinical risk factors, we found that myocardial T2 was independently associated with a reduction in LV mass, suggesting that myocardial edema potentially contributed to LV remodeling during chemotherapy.

This study has several limitations. (1) This single-center study had a relatively small sample size. Nevertheless, the findings demonstrated that T2 mapping can be used to assess myocardial injury in patients undergoing chemotherapy for gynecologic malignancies. Future studies with larger populations are needed to strengthen our findings. (2) Most enrolled patients were at critical condition, meaning that they had to start chemotherapy as soon as possible. Consequently, a baseline CMR examination before chemotherapy was not performed. Given that situation, we recruited healthy volunteers to act as a control group. (3) Because the experiment was conducted during the COVID-19 outbreak, some patients contracted the virus, and thus, the rate of follow-up in the study was low. Future studies with larger cohorts are required to investigate whether myocardial T2 can predict a reduction in LVEF. (4) Our patient cohort received several different chemotherapy regimens. Although the effect of chemotherapy on myocardial edema was adjusted for drug types, the individual effects of the various drugs require further elucidation. (5) This study didn’t measure N-terminal pro-B type natriuretic peptide in patients, the association between N-terminal pro-B type natriuretic peptide and myocardial T2 needs further study with longer follow-up.

## Conclusion

In patients receiving chemotherapy for gynecologic malignancies, myocardial edema develops with the increase of chemotherapy cycles received. The myocardial edema is associated with a reduction in LV mass. T2 mapping allows the assessment of myocardial injury and the monitoring of myocardial edema during chemotherapy.

## Data availability statement

The original contributions presented in the study are included in the article/supplementary material. Further inquiries can be directed to the corresponding author.

## Ethics statement

The clinical protocol of this study was approved by the institutional ethics review board of West China Second Hospital and Chinese Clinical Trial Registry (ChiCTR-DDD-17013450). The patients/participants provided their written informed consent to participate in this study.

## Author contributions

Conceptualization, Y-KG and Z-GY; methodology and formal analysis, M-XY, XL, and LY; data curation and investigation, Q-LL, D-QW, and K-ML; supervision, R-TY and X-JL; original draft, M-XY and XL; review and editing, X-SL, CF, and X-MM; guarantor, Y-KG. All authors contributed to the article and approved the submitted version.

## Funding

This work was supported by National Natural Science Foundation of China (82071874, 81971586, 81771897, 81901712, 81771887,82202094, 82102022, 82120108015); Sichuan Science and Technology Program (2020YFS0050, 2020YJ0029, 2017TD0005, 21ZDYF1967; 2022NSFSC1600,2019YFS0430); Fundamental Research Funds for the Central University (SCU2020D4132); Clinical Research Finding of Chinese Society of Cardiovascular Disease of 2019 (HFCSC2019B01) and 1·3·5 project for disciplines of excellence, West China Hospital, Sichuan University (ZYGD18013, ZYGD18019).

## Conflict of interest

The authors declare that the research was conducted in the absence of any commercial or financial relationships that could be construed as a potential conflict of interest.

## Publisher’s note

All claims expressed in this article are solely those of the authors and do not necessarily represent those of their affiliated organizations, or those of the publisher, the editors and the reviewers. Any product that may be evaluated in this article, or claim that may be made by its manufacturer, is not guaranteed or endorsed by the publisher.

## Abbreviations

CMR, cardiac magnetic resonance; LGE, late gadolinium enhancement; LVEF, left ventricular eject fraction; LVEDV, left ventricular end-diastolic volume; LVESV, left ventricular end-diastolic volume; CI, coefficient interval.
