# Supplementary material for: Myocardial edema during chemotherapy for gynecologic malignancies: A cardiac magnetic resonance T2 mapping study
Source: Front Oncol. 2022 Oct 3;12:961841. doi: 10.3389/fonc.2022.961841 (PMC9574218; doi:10.3389/fonc.2022.961841)
Supplement: Supplementary file 1 [file Table_1.docx]

Table S1. CMR findings between patients and normal controls

| CMR variables | Normal controls  (n = 41) | Patients  (n = 73) | P value |
| --- | --- | --- | --- |
| LVEF, % | 63.10 (58.91 to 66.97) | 62.46 (57.70 to 66.06) | 0.315 |
| Indexed LVEDV, ml/m2 | 69.58 ± 7.97 | 64.44 ± 12.94 | 0.038* |
| Indexed LVESV, ml/m2 | 26.61 ± 5.63 | 25.58 (19.44 to 29.32) | 0.247 |
| Indexed LV mass, g/m2 | 46.90 ± 7.74 | 43.80 ± 8.61 | 0.047* |
| LGE present | 0 (0%) | 17 (23.29%) | 0.001* |

Abbreviations: LV = left ventricular；LVEF = left ventricular eject fraction；LVEDV = left ventricular end-diastolic volume；LVESV = left ventricular end-diastolic volume; LGE = late gadolinium enhancement. *, P < 0.05 vs. normal control.

Table S2. CMR findings in patients between two CMR scans

|  | 1st CMR  (n = 35) | 2nd CMR  (n = 35) | P value |
| --- | --- | --- | --- |
| LVEF, % | 62.91 (59.55 to 65.89) | 63.56 (59.31 to 67.17) | 0.417 |
| Indexed LVEDV, ml/m2 | 67.46 ± 13.81 | 67.58 ± 11.67 | 0.949 |
| Indexed LVESV, ml/m2 | 25.81 ± 7.53 | 25.85 (20.93 to 30.00) | 0.656 |
| Indexed LV mass, g/m2 | 47.17 ± 7.73 | 44.33 ± 8.36 | <0.001* |
| LGE present | 8 (22.86%) | 11 (31.43%) | 0.250 |

Abbreviations as in Table S1. *, P < 0.05 vs. 1st CMR.
